# DARE Train-the-Trainer Pedagogy Development Using 2-Round Delphi Methodology

**DOI:** 10.1155/2016/5460964

**Published:** 2016-08-31

**Authors:** Wei Wei Dayna Yong, Phek Hui Jade Kua, Swee Sung Soon, Pin Pin Maeve Pek, Marcus Eng Hock Ong

**Affiliations:** ^1^Yong Loo Lin School of Medicine, National University of Singapore, Singapore; ^2^Department of Emergency Medicine, KK Women's and Children's Hospital, Singapore; ^3^Department of Pharmacy, National University of Singapore, Singapore; ^4^Department of Emergency Medicine, Singapore General Hospital, Singapore

## Abstract

The Dispatcher-Assisted first REsponder programme aims to equip the public with skills to perform hands-only cardiopulmonary resuscitation (CPR) and to use an automated external defibrillator (AED). By familiarising them with instructions given by a medical dispatcher during an out-of-hospital cardiac arrest call, they will be prepared and empowered to react in an emergency. We aim to formalise curriculum and standardise the way information is conveyed to the participants. A panel of 20 experts were chosen. Using Delphi methodology, selected issues were classified into open-ended and close-ended questions. Consensus for an item was established at a 70% agreement rate within the panel. Questions that had 60%–69% agreement were edited and sent to the panel for another round of voting. After 2 rounds of voting, 70 consensus statements were agreed upon. These covered the following: focus of CPR; qualities and qualifications of trainers; recognition of agonal breathing; head-tilt-chin lift; landmark for chest compression; performance of CPR when injuries are present; trainers' involvement in training lay people; modesty of female patients during CPR; AED usage; content of trainer's manual; addressing of questions and answers; updates-dissemination to trainers and attendance of refresher courses. Recommendations for pedagogy for trainers of dispatcher-assisted CPR programmes were developed.

## 1. Introduction

Out-of-hospital cardiac arrest (OHCA) is one of the main causes of death, of which 65.4% occur at home in Asia [1]. Annually, there are 700,000 cardiac arrest cases in Europe and more than 400,000 cases in America [2]. In Singapore, the annual incidence of OHCA is at least 1,400 cases, of which only 3% survived to discharge [3].

International studies have shown that early cardiopulmonary resuscitation (CPR) improves the chances of survival [4]. Furthermore, a review of several large-scale studies emphasised the importance of dispatcher-assisted CPR in improving bystander CPR and OHCA survival rates [5, 6].

Locally, the bystander CPR rate is only around 20% [3]. Clearly, there is a need to improve these rates which can be done via a local dispatcher-assisted CPR programme. To do so, we conceived a simplified programme for the lay public to learn how to perform effective CPR and use an AED while guided by a medical dispatcher over the telephone.

Traditional CPR classes focus heavily on the rescuer working alone. In our programme, we focus on the rescuer cooperating with the dispatcher. The trainers of this community outreach programme are individuals who are both CPR and AED certified. They guide the participants during the hands-on session, providing constructive feedback and correcting their CPR technique. Currently, there is no formal train-the-trainer curriculum for a dispatcher-assisted CPR training programme. This train-the-trainer model is an established tool used by organisations which gather content from experts to educate trainers pooled from the community, in order to enable them to instruct target audiences. The advantage of such a model is that it can be propagated in the long term by multiple trainers who can disseminate information back to the community in a timely fashion, making this cost-effective and sustainable [7].

As of now, there is no formal pedagogy to train the trainers how to teach the lay population dispatcher-assisted CPR. We aim to write a structured train-the-trainer curriculum to regulate and homogenize the type of information and the way it is conveyed to the participants during the sessions.

## 2. Methods

### 2.1. Setting

The Dispatcher-Assisted first REsponder (DARE) programme is an hour long programme and includes an explanatory video and an instructor-led hands-on session. This is shorter than the usual CPR and AED certification course which spans at least 4 hours and does not include a humorous video. The participants will learn to recognise a cardiac arrest, dial the local emergency number, familiarise themselves with the medical dispatcher's commands, and perform effective CPR on manikins and how to use an AED.

### 2.2. Study Design

To come up with the trainers' curriculum, consensus was gathered using the Delphi approach [8]. This method involves recruiting a panel of experts to answer questions pertaining to the areas of concern.

The study was exempted from institutional review board approval.

### 2.3. Study Participants

A panel of 20 local experts well-versed in cardiopulmonary resuscitation (CPR), from 9 different institutions, was invited to be a part of the study. These included those who are medically trained, who are personally involved in overseeing the current DARE curriculum, and/or who are first-aid instructors. Their areas of expertise varies and include, but are not limited to, emergency medicine, out-of-hospital cardiac arrest, and education.

### 2.4. Study Protocol and Data Collection

First, a pilot group, comprising 4 people who were familiar with DARE or were involved in studies that used the Delphi method, was created. The principal investigator worked with the pilot group to draw up a questionnaire based on literature review and the feedback from trainers. After discussing with the pilot group, we edited the questionnaire and chose to focus on questions related to 10 core areas. These areas are as follows:

1. The focus of general CPR
2. How the train-the-trainer session should be conducted
3. Recognition of cardiac arrest
4. How CPR should be taught
5. Teaching the usage of AED
6. Precourse reading materials
7. Frequently asked questions
8. Trainers' qualities and qualifications
9. Assessment of trainers
10. Continuity of the programme

The expert panel was created and care was taken not to include the panellists from the pilot group.

#### 2.4.1. Round One of Delphi Method

The first-round Delphi questionnaire was distributed to the experts in December 2015 through an online questionnaire portal (SurveyMonkey*™*). They could provide any specific comments perceived to be necessary to drive a primary consensus. The first round was completed after about one month in January 2016. This first questionnaire consisted of dichotomous answers (yes/no), ranking questions, multiple choice questions, and some required open-ended responses. Open-ended responses allowed the experts to give their input, to clarify the interpretation of the question, and to expose common fallacies. Primary consensus was achieved when 70% of respondents were in agreement for dichotomous and multiple choice question.

This cut-off point was used based on previous studies suggesting that a minimum of 70% agreement is needed for validity when using the Delphi method [9–11].

#### 2.4.2. Round  2 of Delphi Method

The expert panel was informed of round one's preliminary results and of their individual comments. Data of which items had obtained consensus and which had not, with the overall agreement percentage obtained by the experts, were presented to the panel. Items that either did not obtain consensus in the first round but had 60–69% agreement or had some ambiguity in phrasing were included in the second questionnaire. Comments and additional options from round one were taken into consideration and included into round two as well. Where possible, the exact phrasing as round one was used. For multiple choice questions with 60–69% agreement and ranking questions, where possible, the questions were converted to yes/no options for clarity.

**Figure 1:**
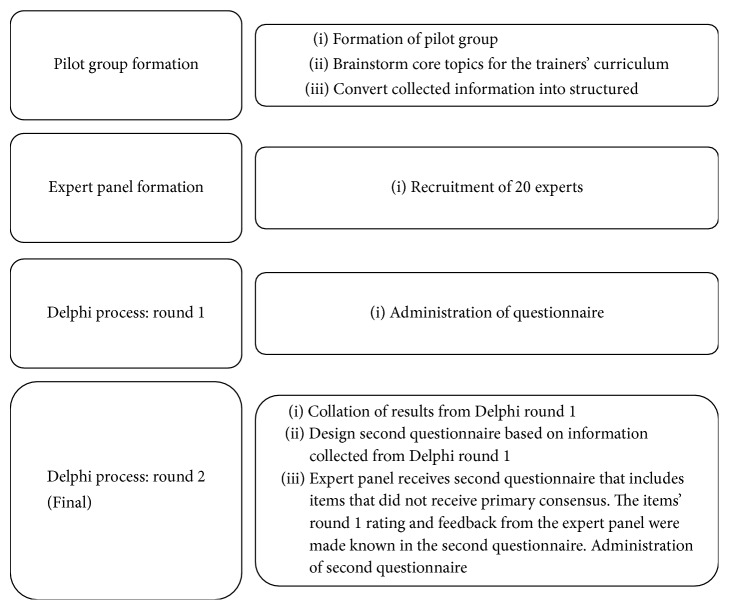
Flow of process.

The second round of Delphi was administered through the previous portal in February 2016 for 3 weeks with the same expert panel. Questions with more than 70% of agreement were regarded as a secondary consensus. We summarized the issue lists from the primary and secondary consensus as the final step by reviewing them via e-mail to establish the recommendations on the curriculum for a dispatcher-assisted train-the-trainer programme. The English language (without translation) was used as the working language in all steps.  Figure 1 is a summary of the process undertaken for the 2-Round Delphi Methodology.

#### 2.4.3. Statistical Analyses

For each item, statistical analysis was performed and the agreement rates were calculated with percentages and frequencies.

## 3. Results

20 experts participated in this study. After opening up the first round of Delphi surveying for 1 month, 25 issues arrived at a consensus. After opening up the second round of Delphi surveying for the same amount of time, an additional 14 issues arrived at a consensus. A total of 70 consensus statements were agreed by the expert panel. No agreement was reached on 11 issues. A summary of the items that received consensus and did not receive consensus can be found in Tables 1 and 2, respectively.

## 4. Discussion

This study is the first of its kind and aims to gather the viewpoints of experts regarding a suitable curriculum for a dispatcher-assisted CPR, train-the-trainer programme. We found that there was 100% agreement on elements that revolved around these core domains:

1. How the train-the-trainer session should be conducted
2. Recognition of cardiac arrest
3. How CPR should be taught
4. Precourse reading materials
5. Frequently asked questions
6. Assessment of trainers

All of the experts agreed that the trainers should correct the hand-positioning of participants when carrying out CPR. The instructors could instruct them of the changes verbally or physically move the participants' hands into the right position. 25% of the experts disagreed that trainers should physically correct the participants' hand position. One of the experts gave feedback that it could be potentially awkward for a male trainer to touch a female participant's hand.

100% of the experts agreed that the curriculum should include a question-and-answer guide so that the trainers will give standardised answers confidently. Common topics that participants in previous DARE training sessions brought up include the need for a good Samaritan law, the risk of being sued, and the fear of breaking ribs during CPR.

The expert panel agreed unanimously that, in assessing the trainers, their ability to conduct the lessons and classroom management skills are important aspects. This suggests that it is not just the theory of resuscitation that should be taught to the trainers but educational methods employing domains including psychology and communication.

Agonal breathing is abnormal breathing that is reported to be present in about 40% of OHCA [12, 13]. It is often confused by bystanders as a sign of life [12, 14] causing CPR to be delayed or withheld, which is associated with poorer outcomes. It was agreed that bystanders' descriptions of agonal breathing should be taught to the trainers. Trainers should be familiar with layman's description of agonal breathing, which could include gasping and noisy breathing [12, 13] and emphasise to lay participants the importance of recognising agonal breathing as a sign of cardiac arrest. They should also dispel any misconception of agonal breathing being a sign of life.

The panel experts all agreed that recognising cardiac arrest, calling 995, and cooperating with the dispatcher for telephone-assisted resuscitation, as well as how to find and use an AED, were important areas that should be included in the curriculum.

Traditionally studies have shown that CPR should not be omitted in the context of a traumatic cardiac arrest [15]. 100% of our experts agreed that the curriculum should specify that CPR be carried out in a victim who has had a fall.

The experts unanimously agreed that the materials given out to the trainees should include current national CPR and AED guidelines. With this material given out before course, it allows the trainers to refresh their memory on the guidelines, reducing unnecessary questions that might be asked during the programme.

## 5. Strengths

The Delphi method was employed in preference to other consensus-achieving methodologies because it is convenient to implement. It is the most time-efficient methodology, as the questionnaires are completed individually, at the expert's own convenience. Consolidation of the results gathered is released for all experts to review, allowing a certain amount of interaction between them [11]. Additionally, this anonymous method [8] eliminates bias resulting from personal status and institutional role in achieving consensus [16].

## 6. Limitations

In this study, there were a few limitations. Firstly, the expert panel was made up of people chosen by invitation to partake in this study. As such, their opinions may not be representative of universal viewpoints. All of the experts were from Singapore. They have worked in Singapore and are familiar with the local resuscitation field. Hence the results might only be relevant in the local setting and should this pedagogy be extended into other settings, certain changes would have to be made or the questionnaire could be redistributed to experts of that specific country. Additionally, the entire study from the administration of the first questionnaire to the closure of the second questionnaire took place within a 2.5 months' period. Within that amount of time, it could be possible that new research in the area might have arisen in the meantime and that the results gathered from this study were overridden by the new research.

## 7. Future Studies

Based on our study, we would like to come up with a train-the-trainer programme that can be launched at a national level. This model appears to be a feasible approach to promote adoption of curricular content on a national scale. Using the results of our study, we can also come up with precourse materials for the trainers to review prior to attending the session. They can even use the materials to revise their knowledge before teaching lay participants.

Future studies can include an audit of both trainers and participants, to see if the execution of the resultant programme is effective. Comparison of the outcomes of the trainers before and after the implementation of the train-the-trainer curriculum should be made. The difference in performance of the trainers after having undergone the current training programme and the new trainer's curriculum should be evaluated as well. Gaps should be addressed and improvements should be made accordingly based on qualitative and quantitative surveys.

## 8. Conclusion

Recommendations for pedagogy for trainers of dispatcher-assisted CPR programmes were developed using the Delphi method. These recommendations should be validated in practical settings.

## Figures and Tables

**Table 1:** List of items that obtained consensus.

Consensus statements showing agreement from Delphi survey rounds 1 and 2			
Consensus statements	Number of respondents in agreement		Established in round
	*N*	%	
*(1) The focus of general CPR*			
(a) Early access is the most important aspect to focus on during the trainers' programme	14	70.0	1
(b) Early CPR is the second most important aspect to focus on during the trainers' programme	15	75.0	1
(c) Early defibrillation is the least important of the three aspects to focus on during the trainers' programme	16	80.0	1

*(2) How the train-the-trainer session should be conducted*			
(a) There should be more than one person (instructor) conducting the session for the trainers	14	70.0	1
(b) There should be hands-on component for the trainers during the session	18	90.0	1
(c) The session should not be conducted entirely through a video for standardisation	14	70.0	1
(d) Qualification an instructor should have to carry out the session			
CPR/AED instructor level	18	94.7	2
CPR/AED trained	17	89.5	2
(e) Video for the train-the-trainer programme should be of a professional tone	17	89.5	2
(f) Video for the train-the-trainer programme should be of a matter-of-fact (factual) tone	16	84.2	2
(g) Video should not cater to English speaking trainers only	16	80.0	1
(h) The video should have both subtitles and voice-over in other languages	14	70.0	1
(i) Hands-on component should be conducted after the video screening	16	80.0	1
(j) During the train-the-trainer session, there should be time allocated for each trainer to role-play with fellow trainers to be the main instructor	17	89.5	2
(k) Topics to be included in the curriculum			
(i) Calling 995 and dispatcher's assistance	19	100.0	2
(ii) The importance of quality CPR/AED	16	84.2	2
(iii) How to carry out CPR and the concerns faced when performing CPR	16	84.2	2
(iv) Spotting common CPR mistakes	16	84.2	2
(v) How to find and use an AED	19	100.0	2
(vi) Material to motivate bystanders to step up and respond to a cardiac arrest	14	73.7	2
(vii) DARE's objectives and effectiveness	17	89.5	2
(viii) Importance of the trainer in DARE	19	100.0	2
(ix) Address the introduction of the CPR card (tells you the depth of chest compression) and MyResponder Application (notifying myResponders to nearby cardiac arrest cases who may render first aid before ambulance arrival)	16	84.2	2
(x) Recognising a cardiac arrest	19	100.0	2
(xi) Trainers' ethics	14	73.7	2

*(3) Key points in recognising a cardiac arrest*			
(a) Bystanders' descriptions of agonal breathing should be taught to the trainers	20	100.0	1
(b) Warning signs like that of lack of breathing should be taught	14	73.7	2
(c) Head-tilt-chin-lift should be covered during the trainers' training session	15	75.0	1
(d) Tapping on the shoulders of a person in possible cardiac arrest should be taught to the trainers	18	90.0	1
(e) Checking for danger should be included in the curriculum	17	85.0	1

*(4) How CPR should be taught *			
(a) Locating the landmark for chest compression: trainers should be taught to position hands in between the nipples	15	78.9	2
(b) The latest American Heart Association updated guidelines stated for rescuers to push hard and fast. However, the guidelines state that compressions should be at least 5 cm but not greater than 6 cm and that chest compressions should be performed at a rate of 100 to 120/min. In a basic resuscitation programme like DARE, the new guidelines should not be taken into consideration and implemented in our train-the-trainer curriculum	15	78.9	2
(c) Trainers should correct the positioning of the DARE lay participants	20	100.0	1
(d) Trainers should physically move the participant's hands into position	15	75.0	1
(e) The same resuscitation method can be taught during the session to be applied to a child in possible cardiac arrest	15	75.0	1
(f) Precautions specific to the paediatric age group should be taught to the trainers	15	75.0	1
(g) CPR should still be instituted to a person in cardiac arrest who had a fall	20	100.0	1
(h) CPR should still be carried out although the patient has a chest injury	18	94.7	2
(i) CPR should still be carried out although the patient has a spinal injury	18	94.7	2
(j) CPR should still be carried out although the patient has bony fractures [rib(s)/limb(s) etc.]	18	94.7	2

*(5) Teaching the usage of AED*			
(a) The person conducting the training session should be using a real AED trainer set to demonstrate how to operate it to the trainers	15	75.0	1
(b) All trainers should practice on a real AED set made for trainers during the session	14	73.7	2
(c) At any one point in time, only 1 person should be operating the trainer AED	15	78.9	1
(d) It is necessary for every trainer to be taught how to use a community AED	16	80.0	1
(e) During training, a real community AED set should be available for the trainers to familiarise themselves with	14	70.0	1
(f) Trainers should be taught where AEDs are found in the community	19	95.0	1
(g) When applying the AED chest pads, the modesty of a female patient should be taken into consideration	16	84.2	2
(h) Her top should be lifted up slightly and not completely to paste the chest pads	16	80.0	1
(i) If she is wearing a dress, the entire dress should not be removed and expose her lower extremities to apply the AED pads	15	75.0	1

*(6) Reading materials catered to the trainers' session*			
(a) Budget should be set aside for the trainers' precourse materials	18	90.0	1
(b) A trainer's manual (to be given out on the training day itself) should be provided for the trainers	18	90.0	1
(c) The trainer's manual should include a DVD of the trainer's video	15	75.0	1
(d) The trainer's manual should include basic CPR and AED guidelines	20	100.0	1
(e) The manual should be translated to cater to non-English speaking trainers	14	70.0	1
(f) There should be an online prelearning component for the trainers prior to attending the training session	15	78.9	2

*(7) Frequently asked questions*			
(a) Trainers should be allowed to answer DARE participants' questions based on their own knowledge	14	70.0	1
(b) There should be a fixed Q&A guidelines for the trainers to refer to and answer from when posed with questions from DARE participants	20	100.0	1
(c) The trainers should be taught to direct all questions to the person conducting the session for the DARE participants that day	14	70.0	1
(d) If participant trainers have any controversial questions during the train-the-trainer's training session, the questions should be collated and answered after a consensus from the DARE coordinators is reached	18	94.7	2
(e) Should participant trainers have any questions during the session, they should approach the DARE coordinators directly during the training	14	73.7	2

*(8) Trainers' qualities and qualifications*			
(a) The minimum qualification a trainer should have before he/she is allowed to sign up to be a trainer for the DARE programme is BCLS and AED certification	14	70.0	1
(b) In view that the DARE programme hopes to reach out to the elderly as well, trainers who are well-versed in dialects should be taught how to teach lay participants in dialects	19	95.0	1
(c) In view of the high dependency on IT equipment to deliver the session, trainers should be taught how to operate IT equipment (i.e., projectors, computers, and basic IT skills)	16	80.0	1
(d) Adolescents (teenagers/students) who are allowed to become a trainer in the future should receive monetary remuneration	17	85.0	1

*(9) Assessment of trainers*			
(a) Trainers should be assessed prior to training to gauge their competency level and suitability	17	85.0	1
(b) Trainers should be required to complete and pass an assessment after the training session before becoming an official DARE trainer	18	90.0	1
(c) Trainers should be assessed on their competency and knowledge in DARE/CPR and AED technique	18	94.7	2
(d) Trainers should be assessed on their ability to conduct the lessons and manage the classroom	19	100.0	2
(e) Trainers should be assessed on their attitude, communication skills, and confidence when teaching	18	94.7	2

*(10) Continuity of the programme *			
(a) E-mailing the trainers is the best platform to disseminate updates to the trainers	16	84.2	2
(b) It is necessary for qualified DARE trainers to attend refresher courses (training session again)	14	70.0	1

Note: total number of responders was 20 for the first round and 19 for the second round.

CPR, cardiopulmonary resuscitation; BCLS, basic cardiac life support; AED, automated external defibrillator; DARE, dispatcher-assisted first responder; Q&A, question and answer; IT, information technology.

**Table 2:** List of items which did not achieve consensus.

Issues on which consensus could not be reached		
Statements	Respondents	
	*N*	%
The instructor who carries out the train-the-trainer session should be a healthcare professional with a current BCLS certificate		
Yes	11	57.9
No	8	42.1

Duration of training session		
2 hours	7	35.0
1.5 hours	4	20.0
1 hour	9	45.0

What should be the maximum number of trainers per session for the train-the-trainer programme? (This is in view that there is only one instructor conducting the programme)		
4 to 6	8	40.0
8 to 10	8	40.0
12 to 20	4	20.0

In view that agonal breathing is reported to be present in about 40% of OHCA, is agonal breathing the only kind of respiration the trainer should be taught to look out for before doing CPR		
Yes	10	50.0
No	10	50.0

Warning signs like that of impending collapse (chest pain, diaphoresis/perspiration, shortness of breath, and drowsiness) should be taught		
Yes	13	68.4
No	6	31.6

Are dispatchers able to teach head-tilt-chin-lift over the phone		
Yes	9	45.0
No	11	55.0

Trainers should be taught to not teach head-tilt-chin-lift to the lay participants		
Yes	8	42.1
No	11	57.9

Materials to motivate trainers attending the trainer's course should be covered in the curriculum		
Yes	12	63.2
No	7	36.8

Importance of maintaining airway should be covered in the curriculum		
Yes	11	57.9
No	8	42.1

How trainers can enhance their communication skills should be covered in the curriculum		
Yes	11	57.9
No	8	42.1

What should the maximum ratio of participant trainers : AED trainer set be		
1 : 1 to 2 : 1	5	26.3
3 : 1 to 4 : 1	11	57.9
5 : 1 to 6 : 1	3	15.8

When applying the AED pads, should the entire bra be removed or just the bra straps be removed		
Entire bra	9	45.0
Bra straps only	11	55.0

Should participant trainers have any questions during the session? They should e-mail the question(s) to an address provided		
Yes	10	52.6
No	9	47.4

Should participant trainers have any questions during the session? They should write the questions down on a piece of paper given		
Yes	12	63.2
No	7	36.8

Should adolescents who are in uniform groups be allowed to be a trainer? (i.e., adolescents whose CCA is NCC/scouts/girl guides/St John's/other uniform groups)		
Yes	12	63.2
No	7	36.8

How often should trainers be updated with new information		
Once a month	0	0.0
Once every 6 months	2	10.5
Once a year	5	26.3
Whenever there are updates	12	63.2
Never	0	0.0

## References

[1] Ong M. E. H., Do Shin S., De Souza N. N. A. (2015). Outcomes for out-of-hospital cardiac arrests across 7 countries in Asia: the Pan Asian Resuscitation Outcomes Study (PAROS). *Resuscitation*.

[2] Sans S., Kesteloot H., Kromhout D. O. (1997). The burden of cardiovascular diseases mortality in Europe. *European Heart Journal*.

[3] Lai H., Choong C. V., Fook-Chong S. (2015). Interventional strategies associated with improvements in survival for out-of-hospital cardiac arrests in Singapore over 10 years. *Resuscitation*.

[4] Valenzuela T. D., Roe D. J., Cretin S., Spaite D. W., Larsen M. P. (1997). Estimating effectiveness of cardiac arrest interventions: a logistic regression survival model. *Circulation*.

[5] Bobrow B. J., Panczyk M., Subido C. (2012). Dispatch-assisted cardiopulmonary resuscitation: the anchor link in the chain of survival. *Current Opinion in Critical Care*.

[6] Culley L. L., Clark J. J., Eisenberg M. S., Larsen M. P. (1991). Dispatcher-assisted telephone CPR: common delays and time standards for delivery. *Annals of Emergency Medicine*.

[7] Orfaly R. A., Frances J. C., Campbell P., Whittemore B., Joly B., Koh H. (2005). Train-the-trainer as an educational model in public health preparedness. *Journal of Public Health Management & Practice*.

[8] Hsu C.-C., Sandford B. A. (2007). The Delphi technique: making sense of consensus. *Practical Assessment, Research & Evaluation*.

[9] Verhagen A. P., De Vet H. C. W., De Bie R. A. (1998). The Delphi list: a criteria list for quality assessment of randomized clinical trials for conducting systematic reviews developed by Delphi consensus. *Journal of Clinical Epidemiology*.

[10] Huisstede B. M. A., Miedema H. S., Verhagen A. P., Koes B. W., Verhaar J. A. N. (2007). Multidisciplinary consensus on the terminology and classification of complaints of the arm, neck and/or shoulder. *Occupational and Environmental Medicine*.

[11] Fernández-Llamazares C. M., Hernández-Gago Y., Pozas M. (2013). Two-round Delphi technique for the consensual design of a paediatric pharmaceutical care model. *Pharmacological Research*.

[12] Bång A., Herlitz J., Martinell S. (2003). Interaction between emergency medical dispatcher and caller in suspected out-of-hospital cardiac arrest calls with focus on agonal breathing. A review of 100 tape recordings of true cardiac arrest cases. *Resuscitation*.

[13] Clark J. J., Larsen M. P., Culley L. L., Graves J. R., Eisenberg M. S. (1992). Incidence of agonal respirations in sudden cardiac arrest. *Annals of Emergency Medicine*.

[14] Rea T. D. (2005). Agonal respirations during cardiac arrest. *Current Opinion in Critical Care*.

[15] Bouillon B., Walther T., Krämer M., Neugebauer E. (1994). Trauma and circulatory arrest. 224 preclinical resuscitations in Cologne in 1987–1990. *Der Anaesthesist*.

[16] Murphy M. K., Black N. A., Lamping D. L. (1998). Consensus development methods, and their use in clinical guideline development. *Health Technology Assessment*.

